# Isolation and characterization of vB_XciM_LucasX, a new jumbo phage that infects *Xanthomonas citri* and *Xanthomonas fuscans*

**DOI:** 10.1371/journal.pone.0266891

**Published:** 2022-04-14

**Authors:** Vinícius Marquioni, Fernando Pacheco Nobre Rossi, Deborah Cezar Mendonça, Layla Farage Martins, Franklin Behlau, João Carlos Setubal, Aline Maria da Silva, Maria Teresa Marques Novo-Mansur

**Affiliations:** 1 Departamento de Genética e Evolução (DGE), Laboratório de Bioquímica e Biologia Molecular Aplicada (LBBMA), Universidade Federal de São Carlos (UFSCar), São Carlos, São Paulo, Brazil; 2 Departamento de Bioquímica, Instituto de Química, Universidade de São Paulo (USP), São Paulo, São Paulo, Brazil; 3 Grupo de Biofísica e Biologia Estrutural “Sérgio Mascarenhas”, Instituto de Física de São Carlos, Universidade de São Paulo (USP), São Carlos, São Paulo, Brazil; 4 Departamento de Pesquisa e Desenvolvimento, Fundo de Defesa da Citricultura (Fundecitrus), Araraquara, São Paulo, Brazil; Dong-A University, REPUBLIC OF KOREA

## Abstract

Citrus canker is one of the main bacterial diseases that affect citrus crops and is caused by *Xanthomonas citri* which affects all citrus species worldwide. New strategies to control citrus canker are necessary and the use of bacteriophages as biocontrol agent could be an alternative. Phages that infect *Xanthomonas* species have been studied, such as XacN1, a myovirus that infects *X*. *citri*. Here we report the isolation and characterization of a new jumbo phage, vb_XciM_LucasX, which infects *X*. *citri* and *X*. *fuscans*. Transmission electron microscopy allowed classification of LucasX in the Myoviridae family, which was corroborated by its genomic sequencing, annotation, and proteome clustering. LucasX has a 305,651 bp-long dsDNA genome. ORF prediction and annotation revealed 157 genes encoding putative structural proteins such as capsid and tail related proteins and phage assembly associated proteins, however, for most of the structural proteins it was not possible assign specific functions. Its genome encodes several proteins related to DNA replication and nucleotide metabolism, five putative RNA polymerases, at least one homing endonuclease mobile element, a terminase large subunit (TerL), an endolysin and many proteins classified as beneficial to the host. Proteome clustering and phylogeny analyses showed that LucasX is a new jumbo phage having as its closest neighbor the *Xanthomonas* jumbo phage Xoo-sp14. LucasX presented a burst size of 40 PFU/infected cell of *X*. *citri* 306, was completely inactivated at temperatures above 50°C, presented survival lower than 25% after 80 s of exposition to artificial UV light and had practically no tolerance to concentrations above 2.5 g/L NaCl or 40% ethanol. LucasX presented optimum pH at 7 and a broad range of *Xanthomonas* hosts, infecting twenty-one of the twenty-three strains tested. Finally, the LucasX yield was dependent on the host strain utilized, resulting one order of magnitude higher in *X*. *fuscans* C 752 than in *X*. *citri* 306, which points out to the possibility of phage yield improvement, an usual challenge for biocontrol purposes.

## 1 Introduction

Citrus canker is one of the main bacterial diseases that affect citrus crops worldwide, along with HLB (huanglongbing) and CVC (citrus variegated chlorosis) [1–3]. The disease includes three main types known as A, B and C, which differ in the symptoms aggressiveness and citrus host range. Citrus canker A, caused by *Xanthomonas citri* subsp. *citri* (also known as *X*. *citri* pv. *citri* A), is the most aggressive form of the disease, affects all citrus species and is found worldwide. Citrus canker B is a milder disease caused by *X*. *fuscans* subsp. *aurantifolii* B and citrus canker C is caused by *X*. *fuscans* subsp. *aurantifolii* C, which has a single citrus host. *X*. *fuscans* B and C were previously classified as *X*. *citri* pv. *aurantifolii* B and C, respectively, and are only found in South America [4]. Citrus trees affected with the disease display characteristic lesions in leaves, fruit and stems, and show a premature loss of diseased leaves and fruit, which decreases citrus productivity [3, 5].

*Xanthomonas citri* is a Gram-negative bacterium belonging to a genus that includes other plant pathogens such as *X*. *oryzae*, *X*. *campestris* and *X*. *vesicatoria* [6]. *X*. *citri* is a rod-shaped bacillus with polar flagella which can be disseminated by the air and the rain and infect susceptible plants through the stomata and wounds on the plant surface [7]. Once inside the host, it can produce the exopolysaccharide xanthan and form a biofilm, which contributes to pathogenicity [8].

The control of citrus canker may be performed using two opposing strategies: i) exclusion of the pathogen, which is based on quarantine measures and the eradication of contaminated and suspect trees, or ii) management of the disease in endemic areas by spraying copper-based bactericides, planting arboreal windbreakers, controlling the citrus leaf miner, and using resistant or less susceptible cultivars. Despite its efficiency on the control of citrus canker, the excessive or misuse of copper may affect the environment and the growth of the citrus root system, leading to the impairment of nutrient uptake and underdevelopment of the tree canopy [9, 10]. Thus, new strategies to control citrus canker are necessary and the use of bacteriophages could be a promising alternative [11]. Phages have been studied as a biocontrol agent for bacterial diseases in many plant species, such as potato, tomato, lettuce, leek and grapes [12–18]. Indeed, phage-based products are now commercially available for the control of pathogenic bacteria affecting crops, livestock and food [19].

Phages that infect *Xanthomonas* species have been isolated and characterized, such as XacN1, a myovirus that infects *X*. *citri* [20] and whose head structure was elucidated by cryo-electron microscopy [21]. With a 390-kbp genome, XacN1 is characterized as a jumbo phage, a designation attributed to those phages with genomes larger than 200 kbp [22]. Phages Cp1 and Cp2 have been traditionally used for the typing of *X*. *citri* strains and their genomic and molecular characterization allowed their assignment to different viral groups [23]. Temperate phages such as XacF1, Cf2 and Xf109 (a *X*. *oryzae* phage) have also been described [24–26]. As of September/2021, however, the NCBI virus genome database (https://www.ncbi.nlm.nih.gov/genomes/GenomesGroup.cgi) includes only 29 *Xanthomonas* phage genomes and several of them were isolated in species other than *X*. *citri*, such as Xf109.

The identification and characterization of new *X*. *citri*-infecting phages could foster their use for biocontrol of citrus canker, as well as increase our understanding of phage diversity and dynamics. In this paper, we report the isolation of LucasX, a new jumbo phage that infects a broad range of *X*. *citri* strains and *X*. *fuscans*, and present its structural and functional features.

## 2 Materials and methods

### 2.1 Bacterial strains and growth conditions

*X*. *citri* and *X*. *fuscans* strains used in this study are listed in Table 1. Throughout this paper, strains belonging to pathotypes A, B, and C are indicated as Xac, XauB and XauC, respectively. The strain used for phage isolation was Xac 306, the first *X*. *citri* strain to be sequenced [27]. Bacterial stocks were kept in 20% glycerol at -80°C. For all experiments, stocks were streaked on Nutrient Agar (NA) medium (Difco NA, BD) and incubated at 30°C for 24–48 h. Single bacterial colonies were grown in Nutrient Broth (NB) medium (Difco NB, BD) at 30°C with shaking at 250 rpm for 24h.

**Table 1:** Susceptibility of *X*. *citri* and *X*. *fuscans* strains to vB_XciM_LucasX.

Species	Strain	Accession number (NCBI)	Susceptibility
**Xac**	Xac 306/12	AE008923.1 (chromosome), AE008925.1 (pXAC64), AE008924.1 (pXAC33)	Yes
	Xac IBSBF 1421/75	-	Yes
	Xac FDC 1301/636	LAUQ00000000.1 (contigs)	Yes
	Xac FDC 1298	-	Yes
	Xac FDC 18	-	Yes
	Xac FDC 22	-	Yes
	Xac FDC 38	-	Yes
	Xac FDC 58	-	Yes
	Xac FDC 118	-	Yes
	Xac FDC 121	-	Yes
	Xac FDC 601	-	Yes
	Xac FDC 604	-	Yes
	Xac FDC 612	-	Yes
	Xac FDC 624	-	Yes
**XauB**	XauB 11122/1631	ACPX00000000.1 (contigs)	Yes
	XauB IBSBF 1583/1565	-	No
	XauB 1566	CP012002.1 (chromosome), CP012003.1 (pXfb35)	No
**XauC**	XauC IBSBF 338/1632	ACPY00000000.1 (contigs)	Yes
	XauC FDC 763	LAUI00000000.1 (contigs)	Yes
	XauC FDC 828	LAUP00000000.1 (contigs)	Yes
	XauC FDC 725	-	Yes
	XauC FDC 535	LAUH00000000.1 (contigs)	Yes
	XauC FDC 752	-	Yes

### 2.2 Phage isolation and propagation

Phages were isolated from a soil sample collected at a citrus grove affected by citrus canker in Matão, São Paulo, Brazil, according to the procedure described by Yoshikawa et al. (2018) [20], with modifications. Six grams of this sample (corresponding to a soil volume of approximately 5 mL) were mixed with NB medium to a final volume of 15 mL and 100 μL of an overnight culture of Xac 306 in a 50-mL tube. The suspension was incubated overnight at 30°C with shaking at 250 rpm and centrifuged at 7,000 *g* for 15 min. The supernatant was filtered through a 0.22-μm-pore membrane attached to a syringe and 100 μL of the filtered solution were mixed with 200 μL of an overnight culture of Xac 306 in 3 mL of semi-solid NA medium (NB medium with 0.4% agar) before plating onto NA medium. After incubation at 30°C overnight, one of the lysis plaques observed was picked up using a Pasteur pipette and diluted in 100 μL of NB medium. The plating procedure was repeated twice to ensure the isolation of a purified sample of the phage (10^9^ PFU/mL in NB medium), which was stored at 4°C.

### 2.3 Transmission electron microscopy (TEM)

An ultrathin carbon film supported by lacey carbon on a copper grid (PELCO) was negatively charged in an easyGlow^TM^ Glow Discharge Cleaning System (PELCO). A volume of 3 μL of the phage sample (10^9^ PFU/mL) was deposited on the grid, followed by 3 μL of water and 3 μL of 2% uranyl acetate. Excess liquid was removed with filter paper. The grid was analyzed in a JEM-2100 (JEOL) at 200 kV.

### 2.4 Genome sequencing, assembly and annotation

DNA was extracted from 2 mL of the phage sample (10^9^ PFU/mL) by a modified phenol-chloroform method. To remove the exopolysaccharide produced by Xac 306, the phage sample was mixed with the same volume of chloroform and 0.7 M NaCl. After centrifugation, the aqueous phase was extracted twice with an equal volume of phenol-chloroform (1:1, v:v) and once with an equal volume of chloroform. The DNA was precipitated with 0.6 volume of isopropanol and 0.1 volume of 3M sodium acetate, washed with 70% ethanol and dissolved in 200 μL of water. This procedure yielded a concentration of ~80 ng/uL and a 260 nm/280 nm absorbance ratio of 1.8 as determined in a NanoVue spectrophotometer (GE).

For whole-genome sequencing, the phage DNA was quantified with the Quant-iT Picogreen dsDNA assay kit (Life Technologies, USA). The shotgun genomic library was prepared using an Illumina Nextera DNA library preparation kit (Illumina, Inc., USA) with 40 ng of DNA. The resulting DNA fragment library was cleaned up with Agencourt AMPure XP beads (Beckman Coulter, Inc., USA). The average fragment size was 665 bp, as determined in a 2100 Bioanalyzer. Quantification of Illumina sequencing library with KAPA Library Quantification Kit, normalization, and sequencing was performed following standard protocols for sequencing in the Illumina MiSeq platform. The library was subjected to one run using the MiSeq Reagent kit v2 (500-cycle format, paired-end reads). On average, Illumina reads from each end of the fragments (R1 and R2) had, respectively, more than 80% and more than 60% of bases with a Phred quality score of at least 30.

Reads quality was visually inspected with FastQC [28]. Since the quality of the R1 reads was higher than that of the R2 reads, only the former was used for genome assembly (~ 2 million reads). Reads were trimmed with Trimmomatic [29] with the following parameters: ILLUMINACLIP:Trimmomatic-0.38/adapters/NexteraPE-PE.fa:2:30:10, LEADING:30, TRAILING:30, SLIDINGWINDOW:4:30, and MINLEN:220. Approximately 920,000 reads (45%) remained after trimming. Contaminant reads, i.e., reads that mapped to Xac 306 genomic or plasmid DNA, were removed with bbduk [30], after which approximately 610,000 reads remained (66%). The genome was then assembled using SPAdes [31]. Finally, the reads were mapped back to the assembled contig using Bowtie2 [32].

The phage genome was initially annotated using the RASTtk pipeline [33] and submitted to the NCBI database (accession number MW825358.1). Phage genome ORF prediction was also performed with PHANOTATE [34], followed by sequences similarity searches using the HMMER3 [35] for profile HMM sequence search against the Viral Orthologs Groups (VOGs; https://vogdb.org/) database (downloaded on August 14, 2021). The tool DeepCapTail [36] was used to predict viral structural proteins (capsid and tail). A linear map of the annotated phage was generated using the DNAFeaturesViewer (https://github.com/Edinburgh-Genome-Foundry/DnaFeaturesViewer) Python library.

### 2.5 Phage genome comparisons and proteome-based clustering

Two phage genome datasets were used for ANI (ANIb and ANI TETRA) analysis using the PyANI tool (https://github.com/widdowquinn/pyani). One dataset consists of all *Xanthomonas* phages RefSeq entries present in the NCBI virus database, and the other one consists of all RefSeq jumbo phages present in both the NCBI virus and the Millard Lab phage databases (http://millardlab.org/bioinformatics/bacteriophage-genomes/phage-genomes-jul2021/). For the proteome clustering analysis the annotated phage genome was analyzed using the vContact2 tool [37] with the complete Millard Lab phage RefSeq database (http://millardlab.org/bioinformatics/bacteriophage-genomes/phage-genomes-jul2021/) consisting of 15,313 phage genomes. Then, the data from the closest related phages were recovered and a new analysis using only them was performed. The resulting protein clusters were evaluated for presence and absence pattern for each grouped phage to generate a heatmap and hierarchical clustering analysis. A core of protein clusters was retrieved, each protein sequence from each phage was aligned, concatenated and the result was used to calculate a maximum likelihood phylogenetic tree using the FastTree [38] with wag model and gamma optimization with 1000 bootstraps. The resulting tree was visualized using the FigTree (https://github.com/rambaut/figtree/releases/tag/v1.4.4) program.

### 2.6 One-step growth curve and phage stability

For the one-step growth curve, cells from 5 mL of an overnight culture of Xac 306 were collected by centrifugation, suspended in 500 μL of NB medium and mixed with 100 μL of a phage sample containing 10^5^ PFU/mL. The starting MOI was 4 x 10^−6^. The mixture was incubated at 30°C for 5 min at 250 rpm to allow the phages to attach to the cells and centrifuged to remove free phages. After the pellet was suspended in 10 mL of NB medium and culture was incubated at 30°C, 100-μL aliquots were taken every 30 min, mixed with 100 μL of Xac 306 from another overnight culture, and plated as described in item 2.2. Since a preliminary assay indicated that cell lysis should occur after 2.5 h, the time between samplings was reduced to 15 min after 2 h of incubation and aliquots were diluted 20 times before the addition of the Xac cells and plating. The experiment was performed in triplicate.

For thermal stability determination, phage samples were incubated in a thermal cycler at temperatures ranging from 30°C to 55°C for 30 min and plated as described above. To determine the phage tolerance to NaCl, phage samples were plated with NaCl added to both solid and semi-solid agar media at concentrations ranging from 0 to 5 g/L. To determine the phage tolerance to UV light, about 135 μL of a phage sample were carefully pipetted onto empty Petri dishes and exposed to UV light of a germicidal lamp in a biosafety cabinet (Aistream Class II Type A2 BSC, Esco). The Petri dishes were placed at 60 cm from the lamp, which has an emission peak at 253.7 nm and the samples were plated as described above after being exposed from 0 min to 2 min 40 sec. For pH stability determination, phage samples were incubated for 30 min in pH values ranging from 3 to 11, diluted and plated as described above. For ethanol tolerance determination, phage samples were incubated for 30 min in ethanol concentrations ranging from 0% to 80%, diluted and plated as described above. All experiments were performed in triplicate.

### 2.7 Host range analysis

The phage host range was determined using the plating method described above and a spot test. Diluted phage samples were plated as described in item 2.2 using Xac 306 and other twenty-two *Xanthomonas* strains belonging to the pathotypes A, B and C (Xac, XauB and XauC) as hosts (Table 1). For the spot test, 2 μL of a phage sample containing 10^9^ PFU/mL were deposited on top of a bacterial streak of those same strains on NA medium. The formation of a clear zone would indicate a susceptible strain. For both experiments, the plates were incubated overnight at 30°C.

### 2.8 Phage yield in different strains

Since strains Xac 636 and XauC 752 produced larger lysis plaques when used as hosts for plating, their phage yields in liquid medium were compared to the yield of the Xac 306 strain, used for the phage isolation. For each strain, an overnight bacterial culture in NB medium was diluted to an optical density at 600 nm of 1.0. A volume of 50 μL of this bacterial sample was mixed with approximately 10^3^ PFU of the phage in 5 mL of NB medium in 50-ml tubes, which were incubated at 30°C and 250 rpm for 24 h. After centrifugation and filtration, the supernatants were diluted and plated using Xac 306 as host for phage quantification. For each strain, the experiment was performed in triplicate.

## 3 Results

### 3.1 Phage isolation and propagation

Using the procedures described above, we have isolated a phage from a soil sample harvested from a grove affected by citrus canker. The phage was isolated and propagated using Xac 306 cells as host in NB medium. Purified phage presented small plaques (~0.7 mm) in 0.4% or 0.3% agar as the top medium. The phage yield was typically around 10^9^ PFU/mL in Xac 306 whereas no phage propagation was detected in this same host cultivated in LB (lysogeny broth).

### 3.2 Phage morphology

**Fig 1:**
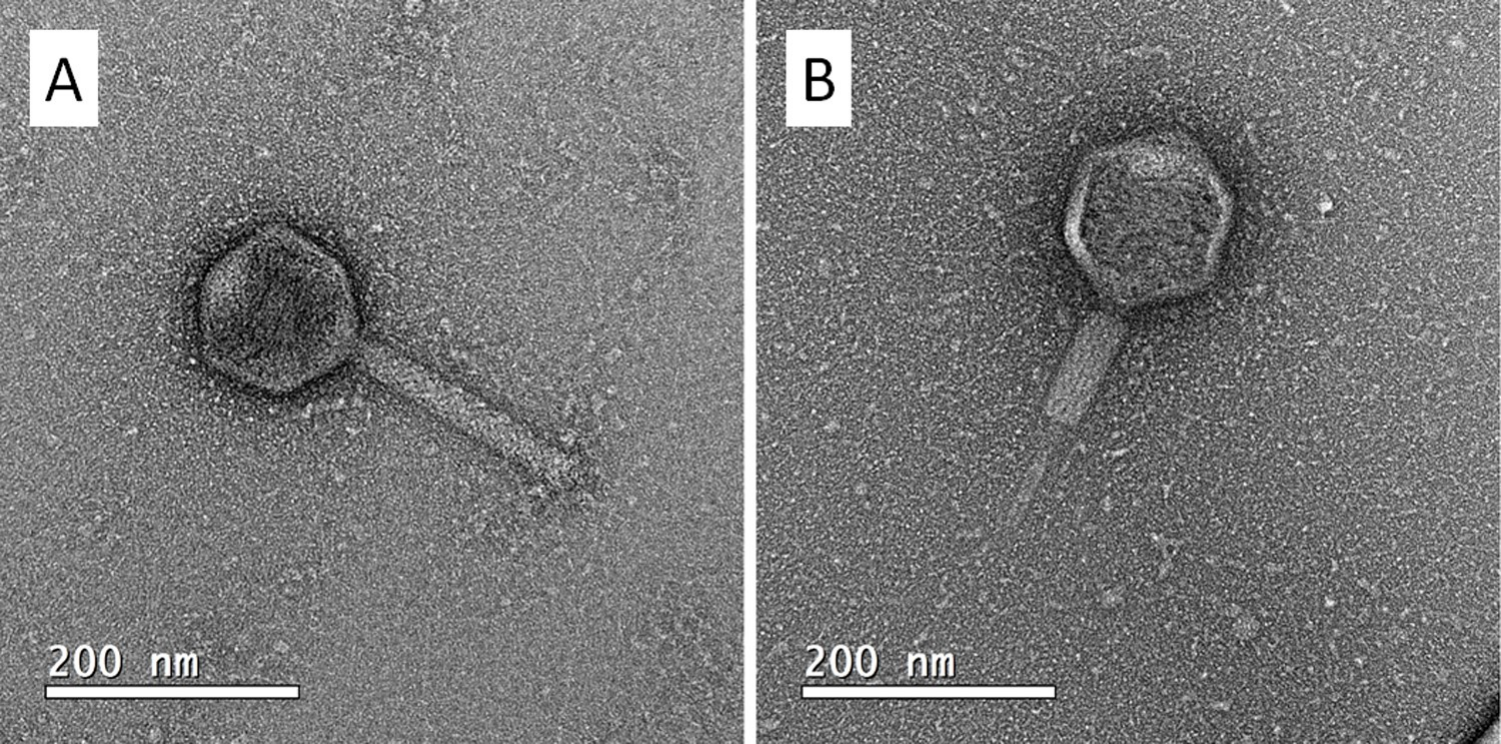
Transmission electron micrograph (TEM) of vB_XciM_LucasX particles. A phage lysate (10^9^ PFU/mL) in NB were placed on a negatively-charged copper grid and stained with 2% uranyl acetate. The grid was analyzed in a JEM-2100 (JEOL) at 200kV. Phage particles can be seen with their tail extended (A) and contracted (B), which is typical of phages of the Myoviridae family.

Phage micrographs obtained by TEM are shown in Fig 1. The phage has an apparently icosahedral head and a contractile tail, with no visible tail fibers, and is approximately 325 nm long when the tail is extended. Based on the presence of a contractile tail and its dsDNA genome, it can be classified in the Myoviridae family.

### 3.3 Phage genomic features and annotation

**Fig 2:**
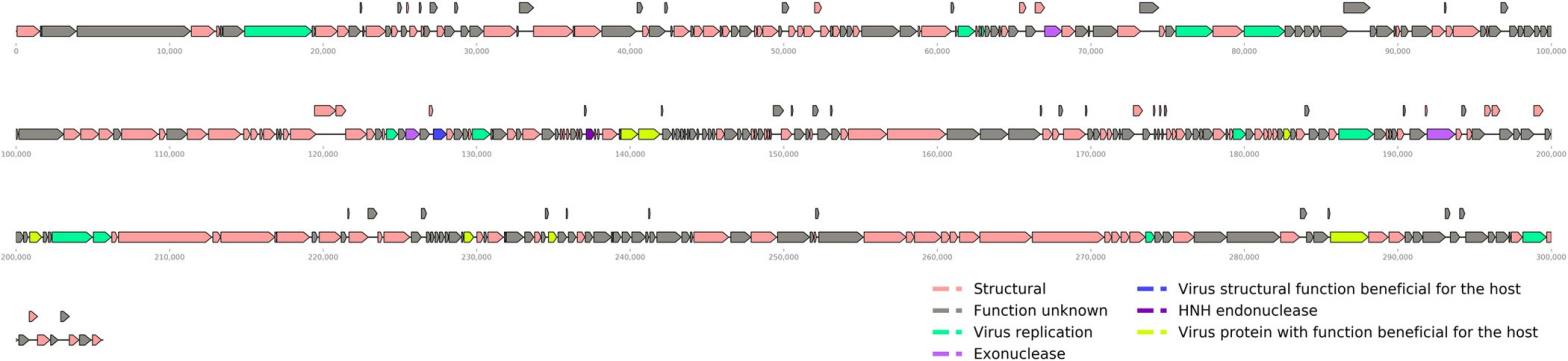
LucasX genome. The annotation was performed by HMM searches using the Viral Ortholog Groups (VOGs) database and the tool DeepCapTail. The figure was produced using the Python library DNAFeaturesViewer.

The genome assembly, performed by SPAdes [31] with ~610,000 reads, produced a longer contig of 305,778 bp, which had no similarity to the genome of the host strain Xac 306. This contig was assumed to be the phage genome and had coverage of 237.7x. The assembly also produced other four contigs shorter than 4,000 bp which mapped to Xac 306 genome. Bowtie2 analysis using all reads (~610,000) indicated that 99.9% of the reads mapped back to the longer contig once, 0.1% did not map, and no reads mapped more than once. The contig had 127 bp direct terminal repeats, indicating that the assembled phage genome was complete. To determine if the ends of the contig corresponded to the physical ends of the genome, we performed a PCR using primers that hybridized near the contig ends and with their 3’ ends pointing outwards. Since the PCR was positive even in the absence of circularization procedures, we hypothesized that the genome can circularize or indeed be either circular or circularly permuted with a length of 305,651 bp with the removal of one of the repeats. A linear map of the annotated phage is shown in Fig 2 and Table 2 shows some of the genomic features of the phage. The GC content of the phage genome was 54.9%, whereas that of the Xac 306 chromosome is 64.8% (Da Silva et al. 2002). Of the 394 ORFs identified by the PHANOTATE tool and searched by HMM, 181 ORFs could be assigned functions, 171 remained as hypothetical, and 42 were identified with no known function. The DeepCapTail analysis identified 157 putative structural proteins, corresponding to about 40% of the ORFs. The HMM analysis identified 1 HNH endonuclease, 7 proteins classified as being beneficial for the host, 12 classified as having virus replication function, and 1 structural protein with beneficial function for the host (S1 Table). Additionally, the hypothetical genes of LucasX were searched against all of the available genes/ proteins of bacteriophages on NCBI virus (database downloaded on July 11, 2021) using BlastP and BlastN. From the 171 CDS predicted to be hypothetical (S1 Table), 149 of them remained as ‘no hit’ for e-values equal to or less than 10^−5^ (S2 Table), indicating that these are novel viral genes since they are not present in other known phages.

**Table 2:** Genomic features of vB_XciM_LucasX.

**Genome size**	305,651 bp
**Sequencing coverage**	237.7x
**GC content**	54.92%
**ORFs**	394
** With function assigned**	181
** Hypothetical**	213
**tRNA genes**	5 (2 Leu, Thr, Ser, Gln)
**Rho-independent terminators**	47

As our results showed that the phage is a novel *X*. *citri*-infecting phage belonging to the Myoviridae family (3.2. and 3.4 sections), it was named vB_XciM_LucasX, according to the nomenclature scheme proposed by Kropinski, Prangishvili, and Lavigne (2009) [39].

### 3.4 Comparative genomic analysis and clustering

The ANIb analysis showed no nucleotide sequence similarity of LucasX with any of the *Xanthomonas* or Jumbo phage genomes publicly available. Using the TETRA nucleotide analysis (ANI TETRA) the higher observed similarity was 69% with the *Xanthomonas* phage Xoo-sp14, a jumbo phage that infects *Xanthomonas oryzae* pv. oryzae [40]. Thus, the ANI analysis confirmed the novelty of the LucasX phage genome.

**Fig 3:**
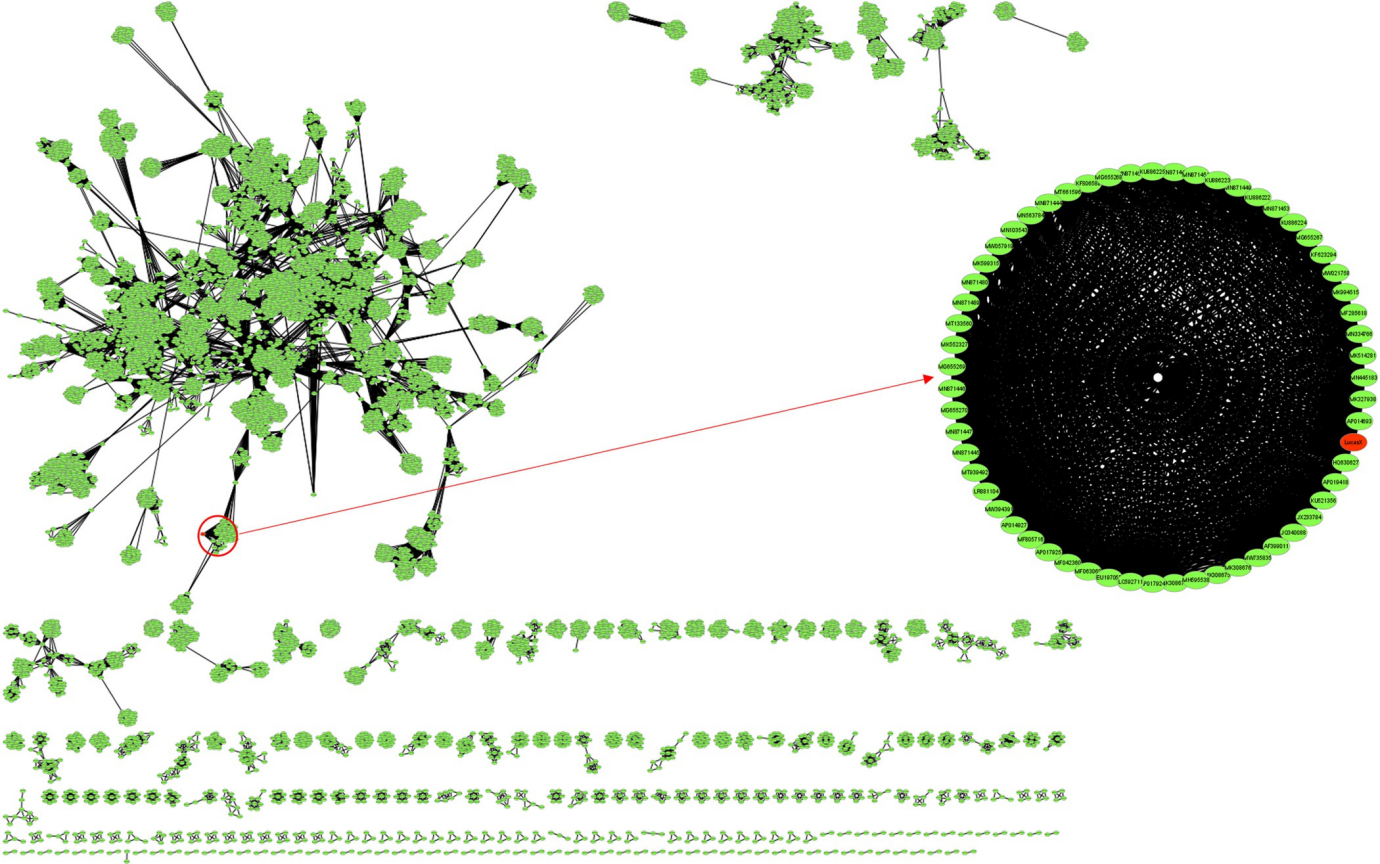
Network analysis based on proteome similarity made with vContact2 and visualized with Cytoscape (v3.8.2). LucasX annotated proteome was assigned to a novel viral cluster (cluster VC_312_0). A total of fifty-eight phages from the Myoviridae family were found to be connected to LucasX, as shown in detail, being that only one of them is not a Jumbo phage. LucasX phage is marked in red.

The proteome clustering and network analysis performed with vContact2 [37] assigned LucasX genome to a novel viral cluster (cluster VC_312_0), connected with other 58 phages from various clusters indicating proteome similarity at family level (Fig 3). This result corroborates with the one obtained by electron microscopy analysis (Fig 1) that evidenced LucasX as a member of the Myoviridae family (S3 Table).

**Fig 4:**
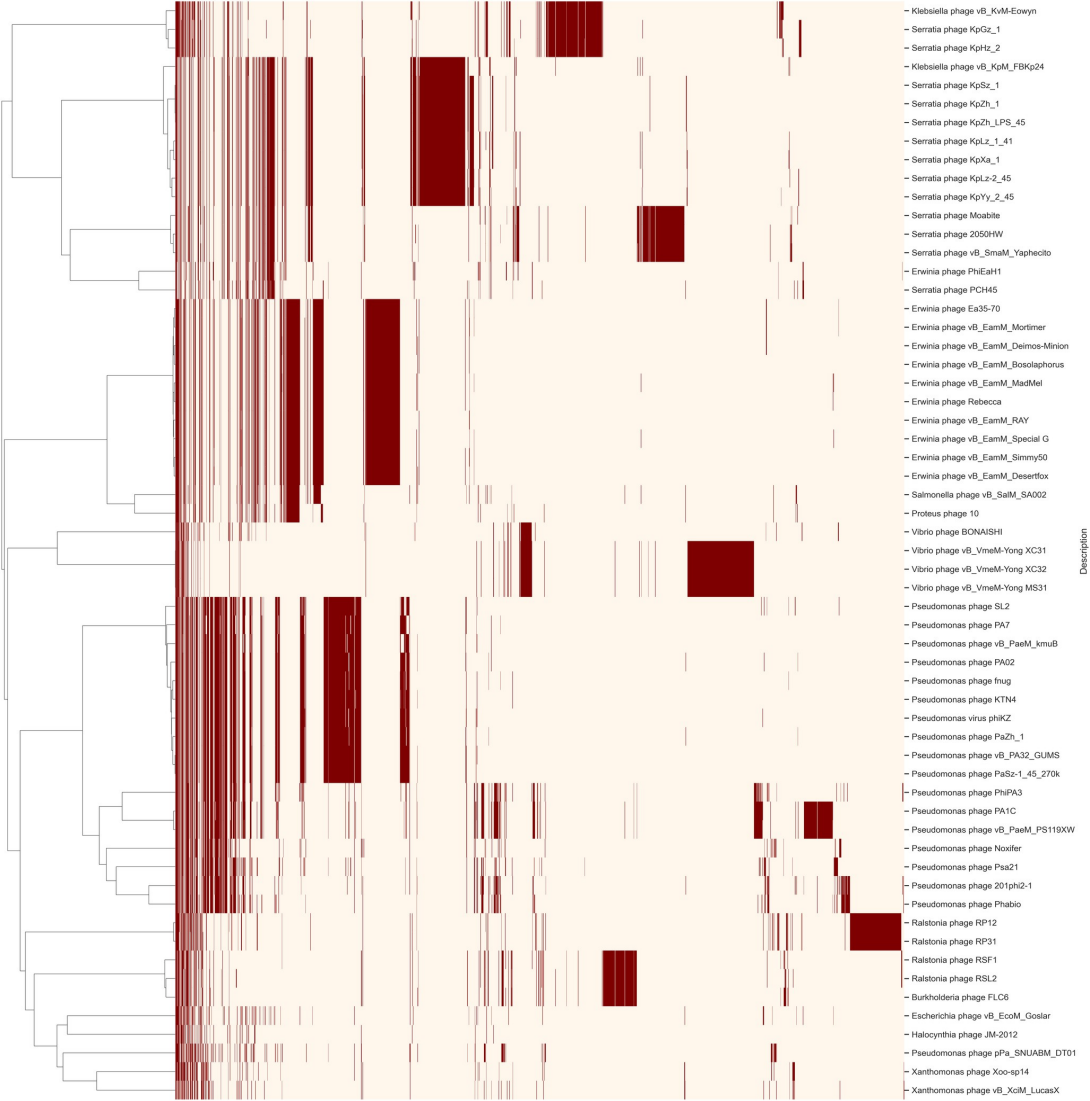
Heatmap analysis of the protein clustering for LucasX and the 58 closest phages performed by vContact2. The presence/absence matrix was generated in python. The red color indicates presence while white represent absence of a protein cluster.

**Fig 5:**
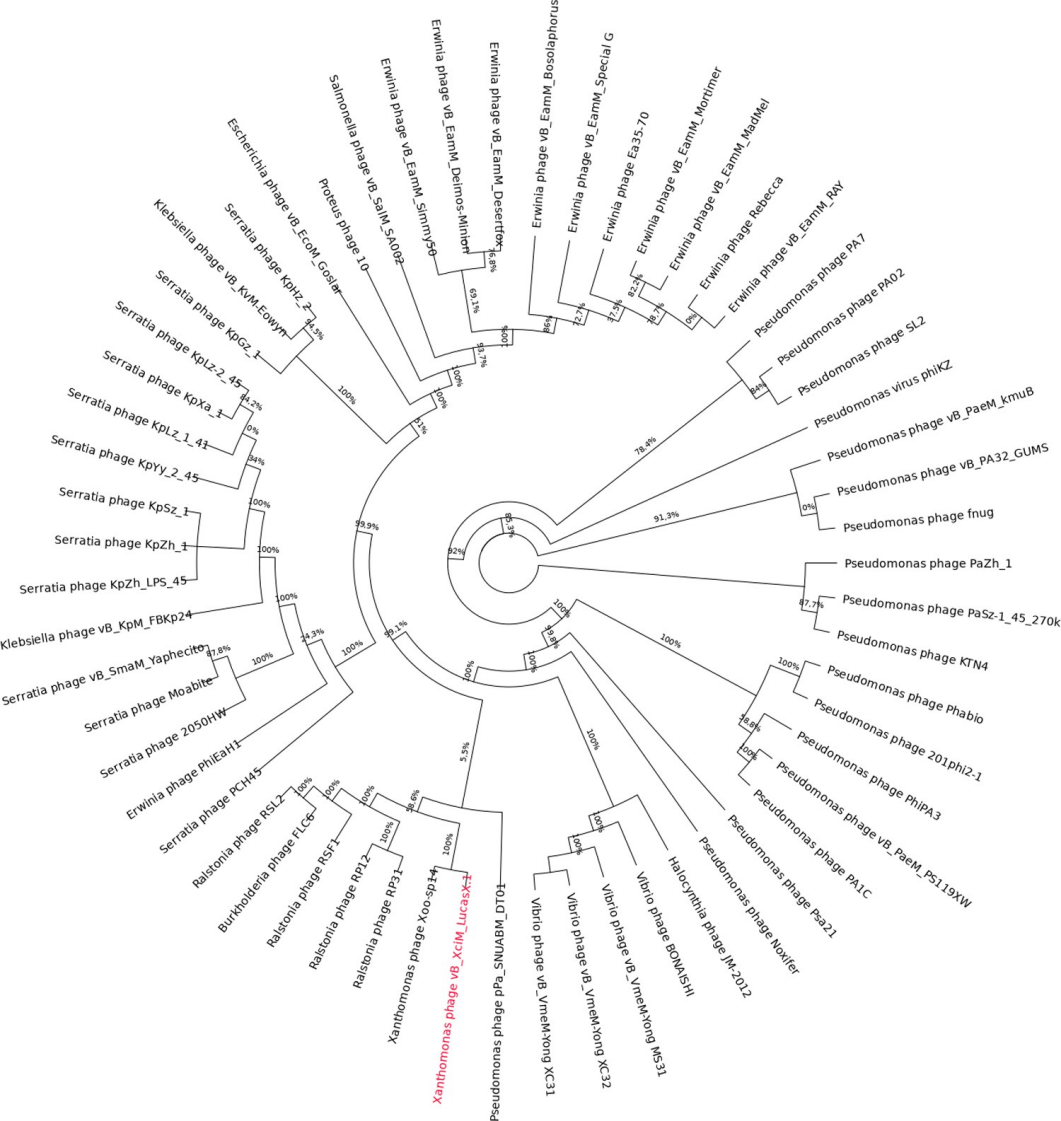
Tree calculated using the core of protein clusters of vB_XciM_LucasX and related phages. The proteome-based comparison places vB_XciM_LucasX (in red) close to other phages of the Myoviridae family. The figure was produced using the FastTree tool for maximum likelihood calculation and the FigTree visualization tool.

Data from the 58 phages, determined as closest to LucasX by the network analysis, were retrieved, including LucasX itself (totaling 59 phages), and a new proteome cluster analysis was performed. The resulting presence and absence matrix showed a highly variable proteome pattern for all analyzed phages (Fig 4). Nevertheless, only the phage Xoo-sp14 showed a closer proteome pattern to LucasX. This analysis also revealed a core of 4 protein clusters in all 59 phages (PC_0000, PC_0001, PC_0002, PC_0003), whose respective annotations are shown in S4 Table. Fig 5 shows the maximum likelihood tree calculated using the core protein clusters, where the closest neighbor of LucasX is the *Xanthomonas* phage Xoo-sp14, as expected from the previous analysis.

### 3.5 Phage growth and stability

**Fig 6:**
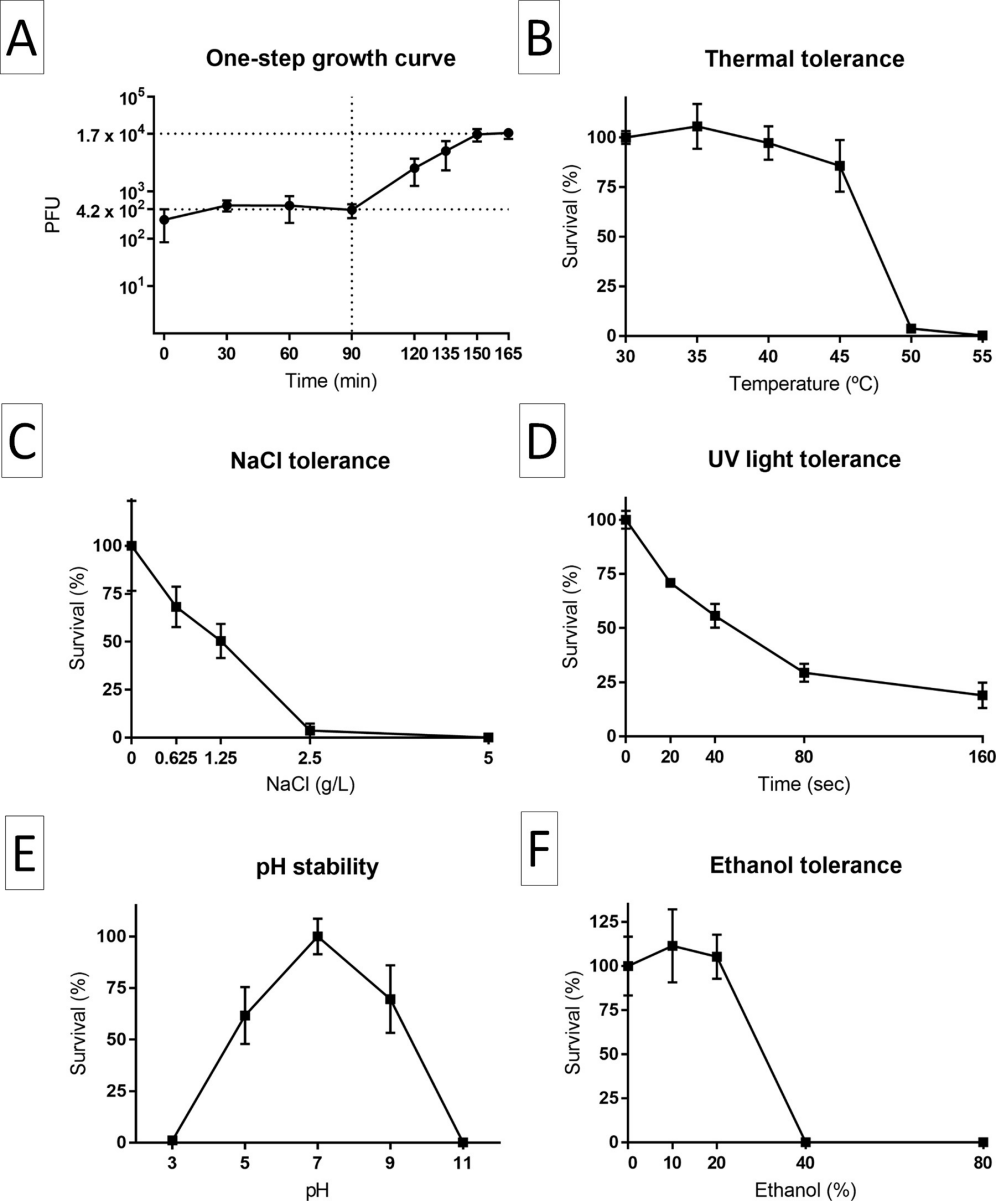
Growth and stability of vB_XciM_LucasX in Xac306. The phage had a latency period of approximately 90 min and a burst size of approximately 40 phages per infected cell of Xac306 (A) and remained viable up to 45°C (B). However, its viability decreased with NaCl concentrations ranging from 0 to 2.5 g/L (C). Its viability also decreased when exposed to the UV light of a safety cabin for a few seconds (D). The optimum pH is 7 and its viability decreased to 60–70% when incubated at pH 5 and 9; it was completely inactivated at pH 3 and 11 (E). Finally, the phage remained stable in ethanol concentrations up to 20% but was inactivated in 40% ethanol (F). The graphs were produced using the software GraphPad Prism and the bars indicate the standard deviation of three replicates.

The results of the growth and stability experiments are shown in Fig 6. LucasX presented a latency period of approximately 90 min and a burst size of approximately 40 PFU/infected cell when Xac 306 was used as host. It remained viable (>80%) up to 45°C but was almost completely inactivated at 50°C for 30 min. LucasX presented a low tolerance to NaCl, with an approximately linear reduction in viability from 100% to 0% in NaCl concentrations ranging from 0 g/L to 2.5 g/L. Viability was reduced to approximately 20% at an apparently exponential rate when LucasX was exposed to UV light for up to 2 min 40 s and to 60% and 70% when it was incubated for 30 min at pH 5 and pH 9, respectively, being that the phage was completely inactivated at pH 3 and 11. Finally, LucasX remained stable in ethanol concentrations up to 20% but was inactivated in 40% ethanol.

### 3.6 Host range

As shown in Table 1, LucasX can infect most of the *Xanthomonas* strains tested as found for both spot test and plating method. All of the fourteen Xac strains and all of the six XauC strains tested were susceptible to phage infection. Of the three XauB strains tested, only one was susceptible (XauB 1631) and the other two were resistant (XauB 1565 and XauB 1566) to phage infection. Except for strains Xac 636 and XauC 752 (which are further discussed in item 3.7), all susceptible strains produce plaques with the same size as those produced by Xac 306.

### 3.7 Phage yield in different strains

**Fig 7:**
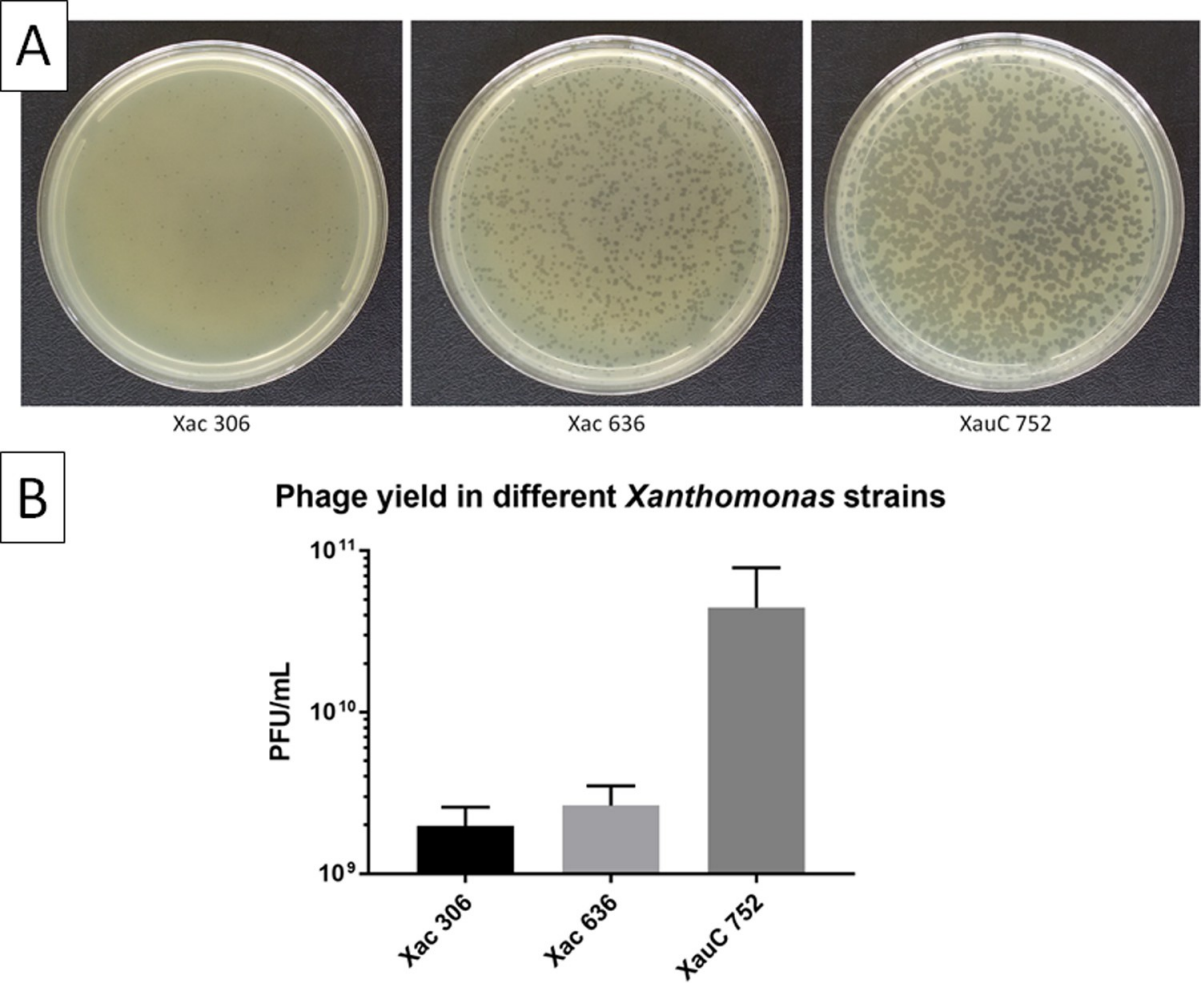
Plaque morphology and phage yield in different *Xanthomonas* strains. Plaques formed by vB_XciM_LucasX on semi-solid NB-agar plates are larger when strains Xac 636 and XauC 752 are used as hosts **(A)**. The plates were photographed after 48 h at 30°C. When propagated in liquid medium, phage yield is slightly higher in Xac 636 and about one order of magnitude higher in XauC 752 relative to Xac 306 **(B)**. The graph was produced using the software GraphPad Prism and the bars indicate the standard deviation of three replicates.

Plating of LucasX using strains Xac 306, Xac 636 and XauC 752 as hosts is shown in Fig 7. The same volume from the same phage sample was used in the three plates. Plaques were visibly larger in Xac 636 (~1.4 mm) and even larger in XauC 752 (~2.1 mm), suggesting that propagation in liquid medium should yield more phages per volume for the later strains. On average, propagation in NB medium (in the conditions described in item 2.8) yielded 1.99 x 10^9^ PFU/mL with Xac 306, 2.64 x 10^9^ PFU/mL with Xac 636, and 4.44 x 10^10^ PFU/mL with XauC 752 (Fig 7).

## 4 Discussion

We have isolated vb_XciM_LucasX, a new Myoviridae jumbo phage that infects *X*. *citri* and *X*. *fuscans* strains, from a soil sample collected at a citrus grove affected with citrus canker in Brazil.

At first, we attempted to isolate phages from soil collected at healthy groves. Although the isolation of *Xanthomonas* phages from soil collected in healthy orchards has been reported [41], our attempts with this kind of sample were not successful. We also tried to isolate phages by mixing the soil samples with NB medium and filtering the samples before inoculating them with Xac 306, but this approach was also unproductive. LucasX was finally isolated by mixing the soil sample (from a citrus grove afflicted with citrus canker) with NB medium and inoculating Xac 306 directly in the suspension, with no prior filtration, as described by Yoshikawa et al. (2018) [20]. Additionally, the same procedure was unsuccessful when LB medium was used instead of NB medium. Our results highlight the importance of using different strategies when isolating phages, especially when exploring new sample sources. Besides, phages have been isolated from unusual environments. Di Lallo et al. (2014) [42] isolated phage ΦPSA2, whose bacterial host is the causal agent of kiwifruit bacterial canker, from a municipal sewage sample. Clavijo-Coppens et al. (2021) [43] have reported that phages able to infect the plant pathogens *Xylella fastidiosa* and *Xanthomonas albilineans* were isolated from surface and sewage waters, but not from infected plants.

After isolating LucasX in NB medium, we were unable to propagate it in LB medium. Since LB has a higher NaCl concentration than NB medium, we studied the LucasX survival in NB-agar with increasing NaCl concentrations. As expected, phage survival decreased as the NaCl concentration was increased. At 5 g/L NaCl, which is the concentration of NaCl in LB medium, phage growth was completely inhibited. These results highlight the importance of exploring different growth media when isolating new phages, since their salt tolerance can vary drastically from those already reported in the literature. Actually, phages have been reported to be typically diluted and stored in SM buffer (saline-magnesium buffer), which contains 5.8 g/L NaCl [44, 45] and an NaCl concentration as high as 1 M (58.44 g/L) has been used for the isolation of *Klebsiella*-infecting phages from coastal water sediments [46]. Korf et al. (2020) [47] have reported that five of six phages under their investigation remained stable for 6 weeks in PBS (phosphate-buffered saline), PBS with 0.1 g/L NaCl, and PBS with 5 g/L NaCl, and even in LB, with no difference in viability among these four conditions.

LucasX has a 305,651 bp-long, dsDNA genome. The ORF prediction using PHANOTATE resulted in a higher number of ORFs than that annotated by RASTtk. Analysis using blast (blast 2.11.0) showed that the vast majority of ORFs identified by the RASTtk were annotated by PHANOTATE and the new ORFs found are overlapping and/or small ORFs. Based on the HMM and DeepCapTail results, the LucasX genome encodes many proteins related to DNA transcription and replication, at least 1 homing endonuclease (LUCX_172) mobile element, many proteins classified as beneficial to the host, as well as enzymes related to nucleotide metabolism. It encodes five putative RNA polymerases (locus tags LUCX_9, LUCX_73, LUCX_76, LUCX_94, LUCX_95) and an endolysin (LUCX_152). ORF LUCX_38 is predicted to encode the terminase large subunit (TerL). Nucleotide metabolism-related proteins include thymidine kinase (LUCX_147), thymidylate synthase (LUCX_157), deoxyuridine 5’-triphosphate nucleotidohydrolase (LUCX_248), ribonucleoside-diphosphate reductase 1 alpha and beta subunits (LUCX_288 and LUCX_289), and dihydrofolate reductase (LUCX_323). Another notable ORF is LUCX_291, which was annotated as a gene transfer agent protein. Gene transfer agents (GTA) are phage-like elements which can form capsids in which random pieces of their genome of origin can be packaged and horizontally transferred [48]. An InterPro analysis with the protein encoded by this ORF indicates the presence of a glycoside hydrolase domain which is also present in the GTA protein found in the bacterium *Rhodobacter capsulatus*, the first organism in which a GTA was identified [49]. Other GTA proteins were not identified in LucasX and the evolutionary significance of this protein remains unknown.

The morphology of LucasX is typical of the Myoviridae family, with an apparently icosahedral head of 125 nm and a contractile tail of approximately 200 nm. The tail sheath could be observed both in extended and in contracted states. When contracted, the sheath is reduced to approximately half of its extended length. No baseplate or spikes were visible by TEM. Most authors employ highly purified phage particles for TEM, usually by PEG precipitation followed by CsCl gradient purification [50, 51], but we were able to obtain sufficiently good micrographs using phage from lysates that had only been filtered. In fact, although samples prepared for TEM will not be biologically active and usually come from a pure sample, it has been proposed that the manipulation of phage samples should be kept to a minimum to avoid changing phage properties [52].

The genome of LucasX has around 157 genes encoding putative structural proteins such as capsid and tail proteins and phage assembly-associated proteins, as depicted in the Fig 2. However, for most of the structural proteins it was not possible assign specific functions so they were annotated as “virion structural protein”. The exceptions are LUCX_16 and LUCX_54, which were annotated as putative chaperone and phage tubulin-like protein, respectively.

Many authors construct phylogenetic trees for phages using specific genes/proteins, such as the major capsid protein, terminases or RNA polymerase [20, 50, 53]. Since LucasX does not show a high degree of similarity to other phages, we decided to use the four protein clusters that were identified as core in the protein cluster analysis. The tree generated by FastTree indicates that LucasX is more closely related to the *Xanthomonas* phage Xoo-sp14 [40] when compared to the other phages in the analysis. The group in which LucasX is found also includes phages that infect *Ralstonia* (phages RSF1, RSL2, RP12 and RP31), *Burkholderia* (phage FLC6), and *Pseudomonas* (phage pPa_SNUABM_DT01).

LucasX has a broad range of *Xanthomonas* hosts, infecting twenty-one of the twenty-three strains tested. Phage XacN1, a *X*. *citri*-infecting jumbo phage, was tested against ten strains, nine of which were susceptible to it [20]. LucasX infected all Xac and XauC strains and also one of the three XauB strains tested. Xac causes the most severe form of citrus canker and is the most widely spread pathotype, whereas XauB is found only in Argentina and is more aggressive on lemon and less aggressive on sweet orange, tangerines and grapefruit [54]. Two XauB strains, XauB 1565 and XauB 1566, were resistant to the infection by LucasX, but their resistance mechanisms remain unknown. As for thermal tolerance, LucasX remained viable up to 45°C but its viability dropped sharply when temperature was increased to 50°C. As for pH stability, LucasX remains at least 60% viable in a pH range of 5 to 9, with a pH optimum of 7. Viability also decreased when the phage was exposed to the UV light of a biosafety cabinet lamp for a few minutes. Although having a broad range of hosts may be advantageous, susceptibility to high temperatures and UV light may be detrimental for biocontrol purposes [55]. Strategies to overcome these limitations have been proposed, such as including skim milk and sucrose in the phage formulation or applying the phages in the evening to reduce exposure to sunlight [56–58]. Nevertheless, LucasX resistance to UV light was assessed in an artificial environment, with direct exposure to a UV lamp, so a lower UV sensibility can be expected in the case of natural sunlight exposure in the field. We also demonstrated that LucasX can be inactivated in 40% ethanol. Our results show that LucasX can be inactivated using ordinary laboratory equipment and chemicals, which may be a relevant biosafety aspect.

After the serendipitous observation that LucasX produced larger plaques when it was plated using strains Xac 636 and XauC 752 as hosts, we hypothesized that propagation in those strains should give higher phage yields. While the yield is only slightly higher in Xac 636, in XauC 752 it is more than one order of magnitude higher in comparison with the strain used for phage isolation. Although this result does not provide strong evidence of a direct relationship between plaque size and yield in liquid-medium propagation, it does show that improvements in phage yield can be obtained by carefully choosing the host strain. The mass production of phages is perceived as one of the biggest challenges to the widespread use of phages as antibacterial agents [59, 60]. Different strategies for improving phage yield have been reported, such as optimizing the starting MOI and bacterial inoculum [61] or using different bioreactor arrangements [62, 63]. To the best of our knowledge, however, there is no report for the improvement of phage yield by systematic screening for high-producing strains of the bacterial host.

## Supporting information

S1 Table LucasX genome annotation. (XLSX)

S2 Table Phage novel genes. (XLSX)

S3 Table Phages with proteomes related to LucasX proteome analyzed in vContact2. (XLSX)

S4 Table Protein clusters found in all 59 phages (LucasX and 58 other phages). (XLSX)
